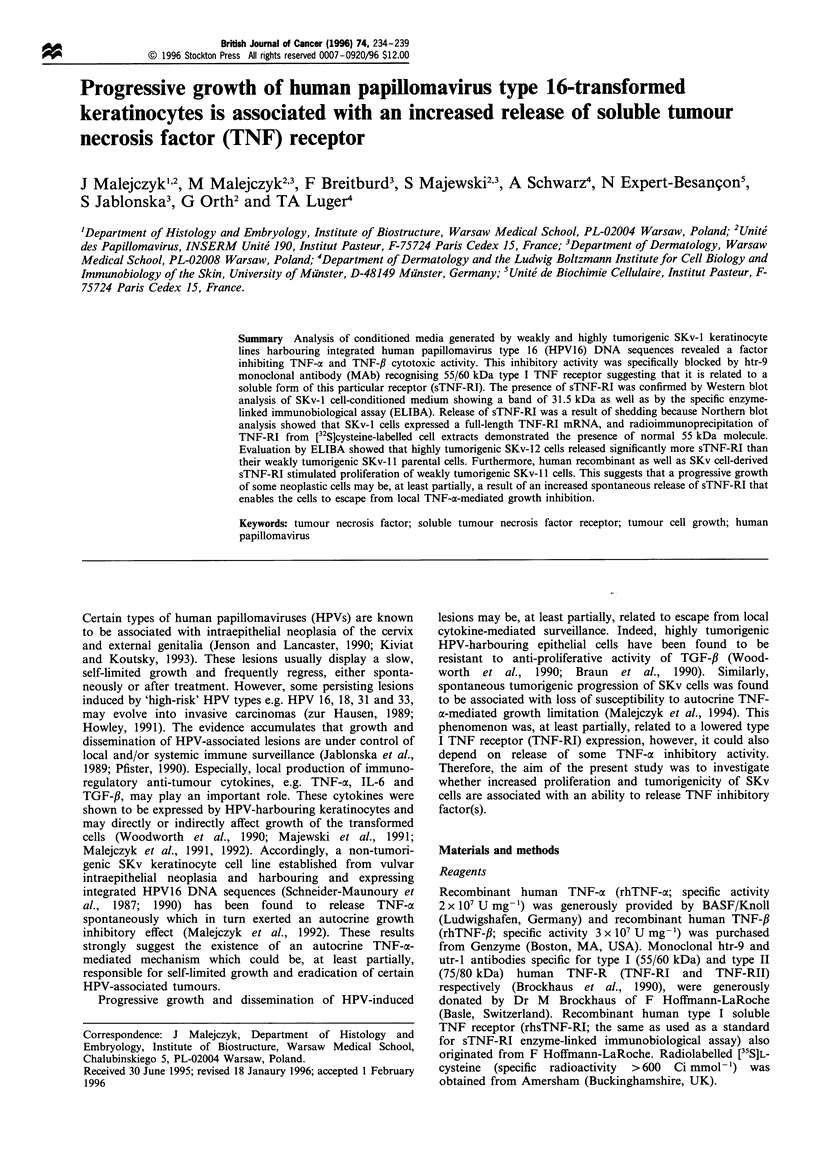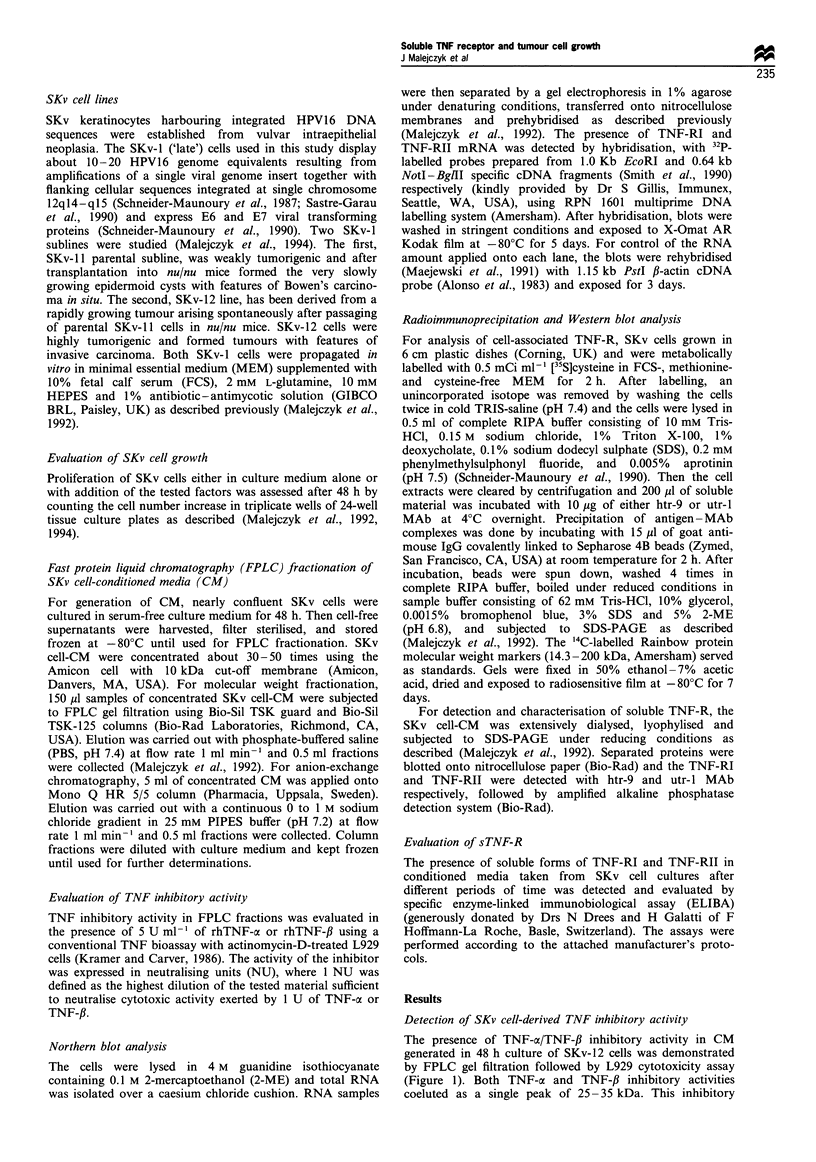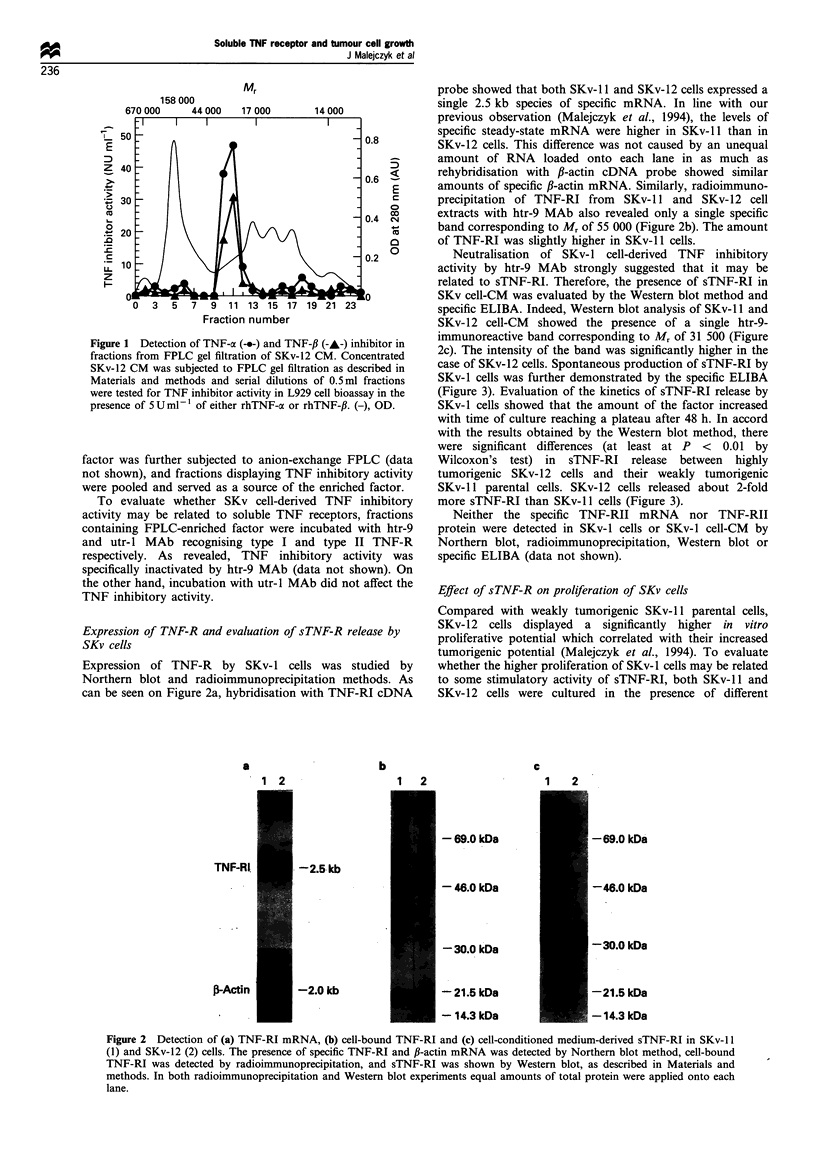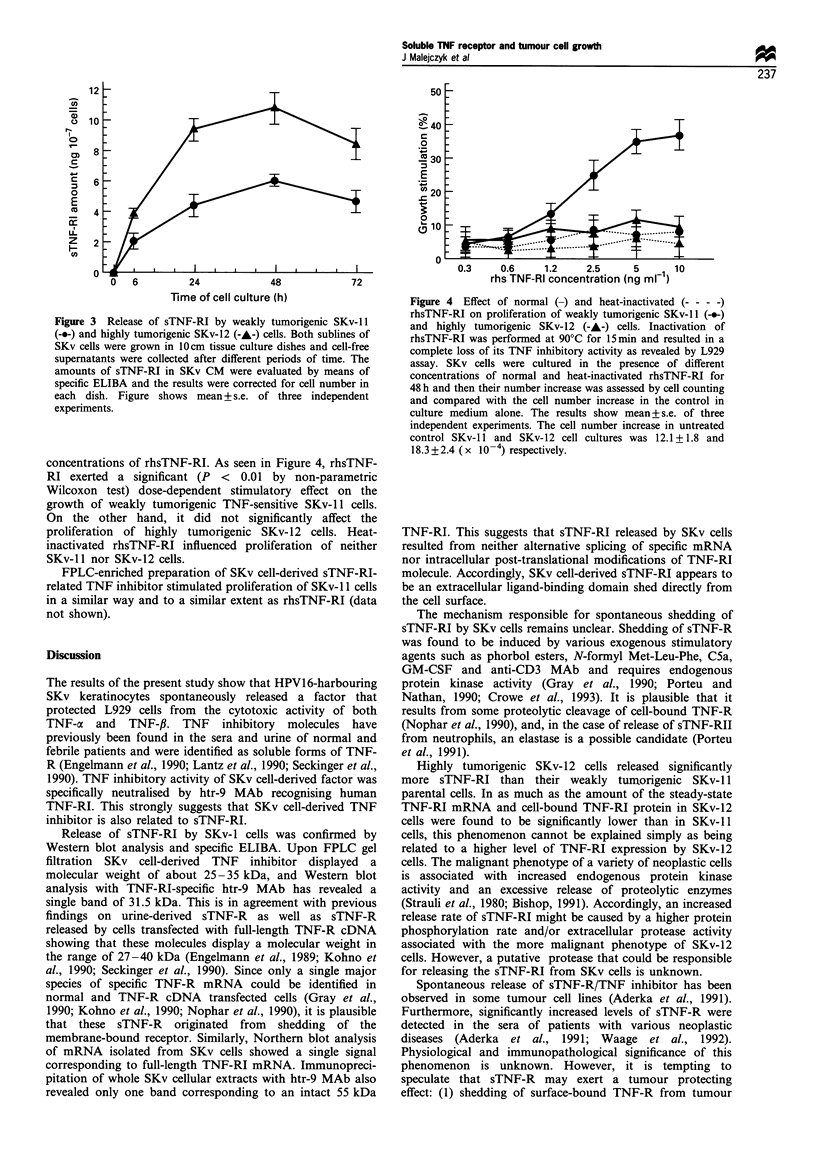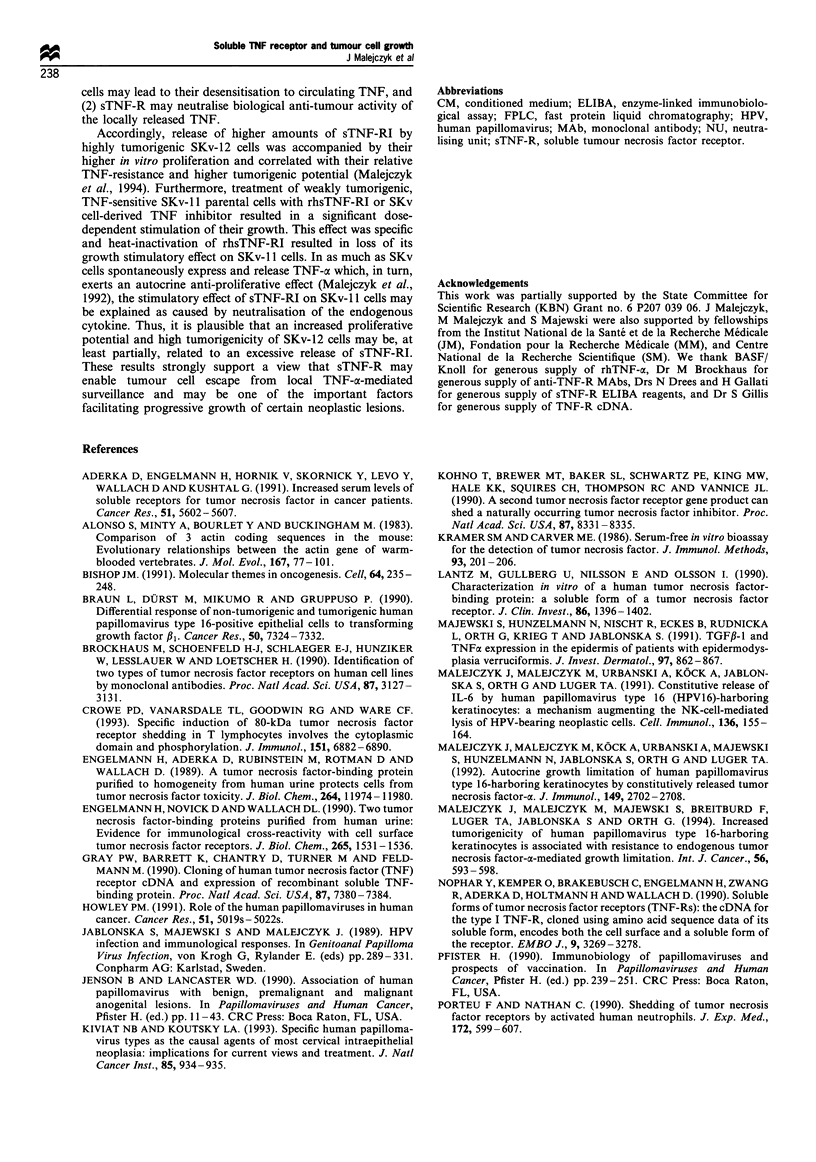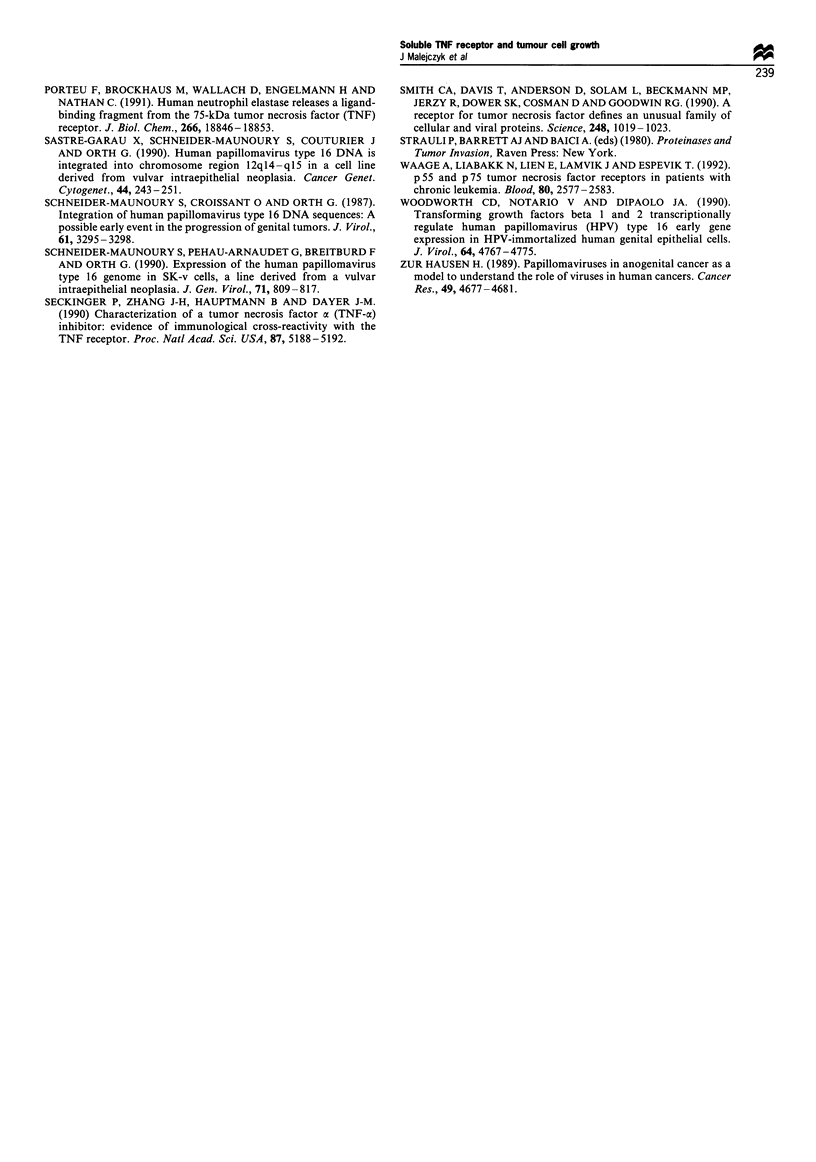# Progressive growth of human papillomavirus type 16-transformed keratinocytes is associated with an increased release of soluble tumour necrosis factor (TNF) receptor.

**DOI:** 10.1038/bjc.1996.343

**Published:** 1996-07

**Authors:** J. Malejczyk, M. Malejczyk, F. Breitburd, S. Majewski, A. Schwarz, N. Expert-Besançon, S. Jablonska, G. Orth, T. A. Luger

**Affiliations:** Department of Histology and Embryology, Warsaw Medical School, Poland.

## Abstract

**Images:**


British Journal of Cancer (1996) 74, 234-239
© 1996 Stockton Press All rights reserved 0007-0920/96 $12.00

### Progressive growth of human papillomavirus type 16-transformed keratinocytes is associated with an increased release of soluble tumour necrosis factor (TNF) receptor

J Malejczyk[1,2], M Malejczyk[2,3], F Breitburd[3], S Majewski[2,3], A Schwarz[4], N Expert-Besançon[5], S Jablonska[3], G Orth[2] and TA Luger[4]

[1]*Department of Histology and Embryology, Institute of Biostructure, Warsaw Medical School, PL-02004 Warsaw, Poland;* [2]*Unité des Papillomavirus, INSERM Unité 190, Institut Pasteur, F-75724 Paris Cedex 15, France;* [3]*Department of Dermatology, Warsaw Medical School, PL-02008 Warsaw, Poland;* [4]*Department of Dermatology and the Ludwig Boltzmann Institute for Cell Biology and Immunobiology of the Skin, University of Münster, D-48149 Münster, Germany;* [5]*Unité de Biochimie Cellulaire, Institut Pasteur, F-75724 Paris Cedex 15, France.*

**Summary** Analysis of conditioned media generated by weakly and highly tumorigenic SKv-1 keratinocyte lines harbouring integrated human papillomavirus type 16 (HPV16) DNA sequences revealed a factor inhibiting TNF-$\alpha$ and TNF-$\beta$ cytotoxic activity. This inhibitory activity was specifically blocked by htr-9 monoclonal antibody (MAb) recognising 55/60 kDa type I TNF receptor suggesting that it is related to a soluble form of this particular receptor (sTNF-RI). The presence of sTNF-RI was confirmed by Western blot analysis of SKv-1 cell-conditioned medium showing a band of 31.5 kDa as well as by the specific enzyme-linked immunobiological assay (ELIBA). Release of sTNF-RI was a result of shedding because Northern blot analysis showed that SKv-1 cells expressed a full-length TNF-RI mRNA, and radioimmunoprecipitation of TNF-RI from [$^{32}$S]cysteine-labelled cell extracts demonstrated the presence of normal 55 kDa molecule. Evaluation by ELIBA showed that highly tumorigenic SKv-12 cells released significantly more sTNF-RI than their weakly tumorigenic SKv-11 parental cells. Furthermore, human recombinant as well as SKv cell-derived sTNF-RI stimulated proliferation of weakly tumorigenic SKv-11 cells. This suggests that a progressive growth of some neoplastic cells may be, at least partially, a result of an increased spontaneous release of sTNF-RI that enables the cells to escape from local TNF-$\alpha$-mediated growth inhibition.

**Keywords:** tumour necrosis factor; soluble tumour necrosis factor receptor; tumour cell growth; human papillomavirus

Certain types of human papillomaviruses (HPVs) are known to be associated with intraepithelial neoplasia of the cervix and external genitalia (Jenson and Lancaster, 1990; Kiviat and Koutsky, 1993). These lesions usually display a slow, self-limited growth and frequently regress, either spontaneously or after treatment. However, some persisting lesions induced by 'high-risk' HPV types e.g. HPV 16, 18, 31 and 33, may evolve into invasive carcinomas (zur Hausen, 1989; Howley, 1991). The evidence accumulates that growth and dissemination of HPV-associated lesions are under control of local and/or systemic immune surveillance (Jablonska *et al.*, 1989; Pfister, 1990). Especially, local production of immunoregulatory anti-tumour cytokines, e.g. TNF-$\alpha$, IL-6 and TGF-$\beta$, may play an important role. These cytokines were shown to be expressed by HPV-harbouring keratinocytes and may directly or indirectly affect growth of the transformed cells (Woodworth *et al.*, 1990; Majewski *et al.*, 1991; Malejczyk *et al.*, 1991, 1992). Accordingly, a non-tumorigenic SKv keratinocyte cell line established from vulvar intraepithelial neoplasia and harbouring and expressing integrated HPV16 DNA sequences (Schneider-Maunoury *et al.*, 1987; 1990) has been found to release TNF-$\alpha$ spontaneously which in turn exerted an autocrine growth inhibitory effect (Malejczyk *et al.*, 1992). These results strongly suggest the existence of an autocrine TNF-$\alpha$-mediated mechanism which could be, at least partially, responsible for self-limited growth and eradication of certain HPV-associated tumours.

Progressive growth and dissemination of HPV-induced lesions may be, at least partially, related to escape from local cytokine-mediated surveillance. Indeed, highly tumorigenic HPV-harbouring epithelial cells have been found to be resistant to anti-proliferative activity of TGF-$\beta$ (Woodworth *et al.*, 1990; Braun *et al.*, 1990). Similarly, spontaneous tumorigenic progression of SKv cells was found to be associated with loss of susceptibility to autocrine TNF-$\alpha$-mediated growth limitation (Malejczyk *et al.*, 1994). This phenomenon was, at least partially, related to a lowered type I TNF receptor (TNF-RI) expression, however, it could also depend on release of some TNF-$\alpha$ inhibitory activity. Therefore, the aim of the present study was to investigate whether increased proliferation and tumorigenicity of SKv cells are associated with an ability to release TNF inhibitory factor(s).

### Materials and methods

### *Reagents*

Recombinant human TNF-$\alpha$ (rhTNF-$\alpha$; specific activity $2 \times 10^7$ U mg$^{-1}$) was generously provided by BASF/Knoll (Ludwigshafen, Germany) and recombinant human TNF-$\beta$ (rhTNF-$\beta$; specific activity $3 \times 10^7$ U mg$^{-1}$) was purchased from Genzyme (Boston, MA, USA). Monoclonal htr-9 and utr-1 antibodies specific for type I (55/60 kDa) and type II (75/80 kDa) human TNF-R (TNF-RI and TNF-RII) respectively (Brockhaus *et al.*, 1990), were generously donated by Dr M Brockhaus of F Hoffmann-LaRoche (Basle, Switzerland). Recombinant human type I soluble TNF receptor (rhsTNF-RI; the same as used as a standard for sTNF-RI enzyme-linked immunobiological assay) also originated from F Hoffmann-LaRoche. Radiolabelled [$^{35}$S]L-cysteine (specific radioactivity >600 Ci mmol$^{-1}$) was obtained from Amersham (Buckinghamshire, UK).

Correspondence: J Malejczyk, Department of Histology and Embryology, Institute of Biostructure, Warsaw Medical School, Chalubinskiego 5, PL-02004 Warsaw, Poland.
Received 30 June 1995; revised 18 Janaury 1996; accepted 1 February 1996

*SKv cell lines*

SKv keratinocytes harbouring integrated HPV16 DNA sequences were established from vulvar intraepithelial neoplasia. The SKv-1 ('late') cells used in this study display about 10–20 HPV16 genome equivalents resulting from amplifications of a single viral genome insert together with flanking cellular sequences integrated at single chromosome 12q14–q15 (Schneider-Maunoury *et al.*, 1987; Sastre-Garau *et al.*, 1990) and express E6 and E7 viral transforming proteins (Schneider-Maunoury *et al.*, 1990). Two SKv-1 sublines were studied (Malejczyk *et al.*, 1994). The first, SKv-11 parental subline, was weakly tumorigenic and after transplantation into *nu/nu* mice formed the very slowly growing epidermoid cysts with features of Bowen's carcinoma *in situ*. The second, SKv-12 line, has been derived from a rapidly growing tumour arising spontaneously after passaging of parental SKv-11 cells in *nu/nu* mice. SKv-12 cells were highly tumorigenic and formed tumours with features of invasive carcinoma. Both SKv-1 cells were propagated *in vitro* in minimal essential medium (MEM) supplemented with 10% fetal calf serum (FCS), 2 mM L-glutamine, 10 mM HEPES and 1% antibiotic–antimycotic solution (GIBCO BRL, Paisley, UK) as described previously (Malejczyk *et al.*, 1992).

*Evaluation of SKv cell growth*

Proliferation of SKv cells either in culture medium alone or with addition of the tested factors was assessed after 48 h by counting the cell number increase in triplicate wells of 24-well tissue culture plates as described (Malejczyk *et al.*, 1992, 1994).

*Fast protein liquid chromatography (FPLC) fractionation of SKv cell-conditioned media (CM)*

For generation of CM, nearly confluent SKv cells were cultured in serum-free culture medium for 48 h. Then cell-free supernatants were harvested, filter sterilised, and stored frozen at −80°C until used for FPLC fractionation. SKv cell-CM were concentrated about 30–50 times using the Amicon cell with 10 kDa cut-off membrane (Amicon, Danvers, MA, USA). For molecular weight fractionation, 150 μl samples of concentrated SKv cell-CM were subjected to FPLC gel filtration using Bio-Sil TSK guard and Bio-Sil TSK-125 columns (Bio-Rad Laboratories, Richmond, CA, USA). Elution was carried out with phosphate-buffered saline (PBS, pH 7.4) at flow rate 1 ml $min^{-1}$ and 0.5 ml fractions were collected (Malejczyk *et al.*, 1992). For anion-exchange chromatography, 5 ml of concentrated CM was applied onto Mono Q HR 5/5 column (Pharmacia, Uppsala, Sweden). Elution was carried out with a continuous 0 to 1 M sodium chloride gradient in 25 mM PIPES buffer (pH 7.2) at flow rate 1 ml $min^{-1}$ and 0.5 ml fractions were collected. Column fractions were diluted with culture medium and kept frozen until used for further determinations.

*Evaluation of TNF inhibitory activity*

TNF inhibitory activity in FPLC fractions was evaluated in the presence of 5 U $ml^{-1}$ of rhTNF-α or rhTNF-β using a conventional TNF bioassay with actinomycin-D-treated L929 cells (Kramer and Carver, 1986). The activity of the inhibitor was expressed in neutralising units (NU), where 1 NU was defined as the highest dilution of the tested material sufficient to neutralise cytotoxic activity exerted by 1 U of TNF-α or TNF-β.

*Northern blot analysis*

The cells were lysed in 4 M guanidine isothiocyanate containing 0.1 M 2-mercaptoethanol (2-ME) and total RNA was isolated over a caesium chloride cushion. RNA samples were then separated by a gel electrophoresis in 1% agarose under denaturing conditions, transferred onto nitrocellulose membranes and prehybridised as described previously (Malejczyk *et al.*, 1992). The presence of TNF-RI and TNF-RII mRNA was detected by hybridisation, with $^{32}P$-labelled probes prepared from 1.0 Kb *Eco*RI and 0.64 kb *Not*I–*Bgl*II specific cDNA fragments (Smith *et al.*, 1990) respectively (kindly provided by Dr S Gillis, Immunex, Seattle, WA, USA), using RPN 1601 multiprime DNA labelling system (Amersham). After hybridisation, blots were washed in stringent conditions and exposed to X-Omat AR Kodak film at −80°C for 5 days. For control of the RNA amount applied onto each lane, the blots were rehybridised (Maejewski *et al.*, 1991) with 1.15 kb *Pst*I β-actin cDNA probe (Alonso *et al.*, 1983) and exposed for 3 days.

*Radioimmunoprecipitation and Western blot analysis*

For analysis of cell-associated TNF-R, SKv cells grown in 6 cm plastic dishes (Corning, UK) and were metabolically labelled with 0.5 mCi $ml^{-1}$ [$^{35}S$]cysteine in FCS-, methionine- and cysteine-free MEM for 2 h. After labelling, an unincorporated isotope was removed by washing the cells twice in cold TRIS-saline (pH 7.4) and the cells were lysed in 0.5 ml of complete RIPA buffer consisting of 10 mM Tris-HCl, 0.15 M sodium chloride, 1% Triton X-100, 1% deoxycholate, 0.1% sodium dodecyl sulphate (SDS), 0.2 mM phenylmethylsulphonyl fluoride, and 0.005% aprotinin (pH 7.5) (Schneider-Maunoury *et al.*, 1990). Then the cell extracts were cleared by centrifugation and 200 μl of soluble material was incubated with 10 μg of either htr-9 or utr-1 MAb at 4°C overnight. Precipitation of antigen–MAb complexes was done by incubating with 15 μl of goat anti-mouse IgG covalently linked to Sepharose 4B beads (Zymed, San Francisco, CA, USA) at room temperature for 2 h. After incubation, beads were spun down, washed 4 times in complete RIPA buffer, boiled under reduced conditions in sample buffer consisting of 62 mM Tris-HCl, 10% glycerol, 0.0015% bromophenol blue, 3% SDS and 5% 2-ME (pH 6.8), and subjected to SDS-PAGE as described (Malejczyk *et al.*, 1992). The $^{14}C$-labelled Rainbow protein molecular weight markers (14.3–200 kDa, Amersham) served as standards. Gels were fixed in 50% ethanol–7% acetic acid, dried and exposed to radiosensitive film at −80°C for 7 days.

For detection and characterisation of soluble TNF-R, the SKv cell-CM was extensively dialysed, lyophylised and subjected to SDS-PAGE under reducing conditions as described (Malejczyk *et al.*, 1992). Separated proteins were blotted onto nitrocellulose paper (Bio-Rad) and the TNF-RI and TNF-RII were detected with htr-9 and utr-1 MAb respectively, followed by amplified alkaline phosphatase detection system (Bio-Rad).

*Evaluation of sTNF-R*

The presence of soluble forms of TNF-RI and TNF-RII in conditioned media taken from SKv cell cultures after different periods of time was detected and evaluated by specific enzyme-linked immunobiological assay (ELIBA) (generously donated by Drs N Drees and H Galatti of F Hoffmann-La Roche, Basle, Switzerland). The assays were performed according to the attached manufacturer's protocols.

### Results

*Detection of SKv cell-derived TNF inhibitory activity*

The presence of TNF-α/TNF-β inhibitory activity in CM generated in 48 h culture of SKv-12 cells was demonstrated by FPLC gel filtration followed by L929 cytotoxicity assay (Figure 1). Both TNF-α and TNF-β inhibitory activities coeluted as a single peak of 25–35 kDa. This inhibitory

**Figure 1** Detection of TNF-$\alpha$ (-●-) and TNF-$\beta$ (-▲-) inhibitor in fractions from FPLC gel filtration of SKv-12 CM. Concentrated SKv-12 CM was subjected to FPLC gel filtration as described in Materials and methods and serial dilutions of 0.5 ml fractions were tested for TNF inhibitor activity in L929 cell bioassay in the presence of 5 U ml$^{-1}$ of either rhTNF-$\alpha$ or rhTNF-$\beta$. (–), OD.

factor was further subjected to anion-exchange FPLC (data not shown), and fractions displaying TNF inhibitory activity were pooled and served as a source of the enriched factor.

To evaluate whether SKv cell-derived TNF inhibitory activity may be related to soluble TNF receptors, fractions containing FPLC-enriched factor were incubated with htr-9 and utr-1 MAb recognising type I and type II TNF-R respectively. As revealed, TNF inhibitory activity was specifically inactivated by htr-9 MAb (data not shown). On the other hand, incubation with utr-1 MAb did not affect the TNF inhibitory activity.

*Expression of TNF-R and evaluation of sTNF-R release by SKv cells*

Expression of TNF-R by SKv-1 cells was studied by Northern blot and radioimmunoprecipitation methods. As can be seen on Figure 2a, hybridisation with TNF-RI cDNA probe showed that both SKv-11 and SKv-12 cells expressed a single 2.5 kb species of specific mRNA. In line with our previous observation (Malejczyk *et al.*, 1994), the levels of specific steady-state mRNA were higher in SKv-11 than in SKv-12 cells. This difference was not caused by an unequal amount of RNA loaded onto each lane in as much as rehybridisation with $\beta$-actin cDNA probe showed similar amounts of specific $\beta$-actin mRNA. Similarly, radioimmunoprecipitation of TNF-RI from SKv-11 and SKv-12 cell extracts with htr-9 MAb also revealed only a single specific band corresponding to $M_r$ of 55 000 (Figure 2b). The amount of TNF-RI was slightly higher in SKv-11 cells.

Neutralisation of SKv-1 cell-derived TNF inhibitory activity by htr-9 MAb strongly suggested that it may be related to sTNF-RI. Therefore, the presence of sTNF-RI in SKv cell-CM was evaluated by the Western blot method and specific ELIBA. Indeed, Western blot analysis of SKv-11 and SKv-12 cell-CM showed the presence of a single htr-9-immunoreactive band corresponding to $M_r$ of 31 500 (Figure 2c). The intensity of the band was significantly higher in the case of SKv-12 cells. Spontaneous production of sTNF-RI by SKv-1 cells was further demonstrated by the specific ELIBA (Figure 3). Evaluation of the kinetics of sTNF-RI release by SKv-1 cells showed that the amount of the factor increased with time of culture reaching a plateau after 48 h. In accord with the results obtained by the Western blot method, there were significant differences (at least at $P < 0.01$ by Wilcoxon's test) in sTNF-RI release between highly tumorigenic SKv-12 cells and their weakly tumorigenic SKv-11 parental cells. SKv-12 cells released about 2-fold more sTNF-RI than SKv-11 cells (Figure 3).

Neither the specific TNF-RII mRNA nor TNF-RII protein were detected in SKv-1 cells or SKv-1 cell-CM by Northern blot, radioimmunoprecipitation, Western blot or specific ELIBA (data not shown).

*Effect of sTNF-R on proliferation of SKv cells*

Compared with weakly tumorigenic SKv-11 parental cells, SKv-12 cells displayed a significantly higher *in vitro* proliferative potential which correlated with their increased tumorigenic potential (Malejczyk *et al.*, 1994). To evaluate whether the higher proliferation of SKv-1 cells may be related to some stimulatory activity of sTNF-RI, both SKv-11 and SKv-12 cells were cultured in the presence of different

**Figure 2** Detection of (**a**) TNF-RI mRNA, (**b**) cell-bound TNF-RI and (**c**) cell-conditioned medium-derived sTNF-RI in SKv-11 (1) and SKv-12 (2) cells. The presence of specific TNF-RI and $\beta$-actin mRNA was detected by Northern blot method, cell-bound TNF-RI was detected by radioimmunoprecipitation, and sTNF-RI was shown by Western blot, as described in Materials and methods. In both radioimmunoprecipitation and Western blot experiments equal amounts of total protein were applied onto each lane.

**Figure 3** Release of sTNF-RI by weakly tumorigenic SKv-11 (-•-) and highly tumorigenic SKv-12 (-▲-) cells. Both sublines of SKv cells were grown in 10 cm tissue culture dishes and cell-free supernatants were collected after different periods of time. The amounts of sTNF-RI in SKv CM were evaluated by means of specific ELIBA and the results were corrected for cell number in each dish. Figure shows mean ± s.e. of three independent experiments.

**Figure 4** Effect of normal (–) and heat-inactivated (- - - -) rhsTNF-RI on proliferation of weakly tumorigenic SKv-11 (-•-) and highly tumorigenic SKv-12 (-▲-) cells. Inactivation of rhsTNF-RI was performed at 90°C for 15 min and resulted in a complete loss of its TNF inhibitory activity as revealed by L929 assay. SKv cells were cultured in the presence of different concentrations of normal and heat-inactivated rhsTNF-RI for 48 h and then their number increase was assessed by cell counting and compared with the cell number increase in the control in culture medium alone. The results show mean ± s.e. of three independent experiments. The cell number increase in untreated control SKv-11 and SKv-12 cell cultures was 12.1 ± 1.8 and 18.3 ± 2.4 ($\times$ 10$^{-4}$) respectively.

concentrations of rhsTNF-RI. As seen in Figure 4, rhsTNF-RI exerted a significant ($P$ < 0.01 by non-parametric Wilcoxon test) dose-dependent stimulatory effect on the growth of weakly tumorigenic TNF-sensitive SKv-11 cells. On the other hand, it did not significantly affect the proliferation of highly tumorigenic SKv-12 cells. Heat-inactivated rhsTNF-RI influenced proliferation of neither SKv-11 nor SKv-12 cells.

FPLC-enriched preparation of SKv cell-derived sTNF-RI-related TNF inhibitor stimulated proliferation of SKv-11 cells in a similar way and to a similar extent as rhsTNF-RI (data not shown).

### Discussion

The results of the present study show that HPV16-harbouring SKv keratinocytes spontaneously released a factor that protected L929 cells from the cytotoxic activity of both TNF-α and TNF-β. TNF inhibitory molecules have previously been found in the sera and urine of normal and febrile patients and were identified as soluble forms of TNF-R (Engelmann *et al.*, 1990; Lantz *et al.*, 1990; Seckinger *et al.*, 1990). TNF inhibitory activity of SKv cell-derived factor was specifically neutralised by htr-9 MAb recognising human TNF-RI. This strongly suggests that SKv cell-derived TNF inhibitor is also related to sTNF-RI.

Release of sTNF-RI by SKv-1 cells was confirmed by Western blot analysis and specific ELIBA. Upon FPLC gel filtration SKv cell-derived TNF inhibitor displayed a molecular weight of about 25–35 kDa, and Western blot analysis with TNF-RI-specific htr-9 MAb has revealed a single band of 31.5 kDa. This is in agreement with previous findings on urine-derived sTNF-R as well as sTNF-R released by cells transfected with full-length TNF-R cDNA showing that these molecules display a molecular weight in the range of 27–40 kDa (Engelmann *et al.*, 1989; Kohno *et al.*, 1990; Seckinger *et al.*, 1990). Since only a single major species of specific TNF-R mRNA could be identified in normal and TNF-R cDNA transfected cells (Gray *et al.*, 1990; Kohno *et al.*, 1990; Nophar *et al.*, 1990), it is plausible that these sTNF-R originated from shedding of the membrane-bound receptor. Similarly, Northern blot analysis of mRNA isolated from SKv cells showed a single signal corresponding to full-length TNF-RI mRNA. Immunoprecipitation of whole SKv cellular extracts with htr-9 MAb also revealed only one band corresponding to an intact 55 kDa TNF-RI. This suggests that sTNF-RI released by SKv cells resulted from neither alternative splicing of specific mRNA nor intracellular post-translational modifications of TNF-RI molecule. Accordingly, SKv cell-derived sTNF-RI appears to be an extracellular ligand-binding domain shed directly from the cell surface.

The mechanism responsible for spontaneous shedding of sTNF-RI by SKv cells remains unclear. Shedding of sTNF-R was found to be induced by various exogenous stimulatory agents such as phorbol esters, *N*-formyl Met-Leu-Phe, C5a, GM-CSF and anti-CD3 MAb and requires endogenous protein kinase activity (Gray *et al.*, 1990; Porteu and Nathan, 1990; Crowe *et al.*, 1993). It is plausible that it results from some proteolytic cleavage of cell-bound TNF-R (Nophar *et al.*, 1990), and, in the case of release of sTNF-RII from neutrophils, an elastase is a possible candidate (Porteu *et al.*, 1991).

Highly tumorigenic SKv-12 cells released significantly more sTNF-RI than their weakly tumorigenic SKv-11 parental cells. In as much as the amount of the steady-state TNF-RI mRNA and cell-bound TNF-RI protein in SKv-12 cells were found to be significantly lower than in SKv-11 cells, this phenomenon cannot be explained simply as being related to a higher level of TNF-RI expression by SKv-12 cells. The malignant phenotype of a variety of neoplastic cells is associated with increased endogenous protein kinase activity and an excessive release of proteolytic enzymes (Strauli *et al.*, 1980; Bishop, 1991). Accordingly, an increased release rate of sTNF-RI might be caused by a higher protein phosphorylation rate and/or extracellular protease activity associated with the more malignant phenotype of SKv-12 cells. However, a putative protease that could be responsible for releasing the sTNF-RI from SKv cells is unknown.

Spontaneous release of sTNF-R/TNF inhibitor has been observed in some tumour cell lines (Aderka *et al.*, 1991). Furthermore, significantly increased levels of sTNF-R were detected in the sera of patients with various neoplastic diseases (Aderka *et al.*, 1991; Waage *et al.*, 1992). Physiological and immunopathological significance of this phenomenon is unknown. However, it is tempting to speculate that sTNF-R may exert a tumour protecting effect: (1) shedding of surface-bound TNF-R from tumour cells may lead to their desensitisation to circulating TNF, and (2) sTNF-R may neutralise biological anti-tumour activity of the locally released TNF.

Accordingly, release of higher amounts of sTNF-RI by highly tumorigenic SKv-12 cells was accompanied by their higher *in vitro* proliferation and correlated with their relative TNF-resistance and higher tumorigenic potential (Malejczyk *et al*., 1994). Furthermore, treatment of weakly tumorigenic, TNF-sensitive SKv-11 parental cells with rhsTNF-RI or SKv cell-derived TNF inhibitor resulted in a significant dose-dependent stimulation of their growth. This effect was specific and heat-inactivation of rhsTNF-RI resulted in loss of its growth stimulatory effect on SKv-11 cells. In as much as SKv cells spontaneously express and release TNF-α which, in turn, exerts an autocrine anti-proliferative effect (Malejczyk *et al*., 1992), the stimulatory effect of sTNF-RI on SKv-11 cells may be explained as caused by neutralisation of the endogenous cytokine. Thus, it is plausible that an increased proliferative potential and high tumorigenicity of SKv-12 cells may be, at least partially, related to an excessive release of sTNF-RI. These results strongly support a view that sTNF-R may enable tumour cell escape from local TNF-α-mediated surveillance and may be one of the important factors facilitating progressive growth of certain neoplastic lesions.

**Abbreviations**

CM, conditioned medium; ELIBA, enzyme-linked immunobiological assay; FPLC, fast protein liquid chromatography; HPV, human papillomavirus; MAb, monoclonal antibody; NU, neutralising unit; sTNF-R, soluble tumour necrosis factor receptor.

**Acknowledgements**

This work was partially supported by the State Committee for Scientific Research (KBN) Grant no. 6 P207 039 06. J Malejczyk, M Malejczyk and S Majewski were also supported by fellowships from the Institut National de la Santé et de la Recherche Médicale (JM), Fondation pour la Recherche Médicale (MM), and Centre National de la Recherche Scientifique (SM). We thank BASF/Knoll for generous supply of rhTNF-α, Dr M Brockhaus for generous supply of anti-TNF-R MAbs, Drs N Drees and H Gallati for generous supply of sTNF-R ELIBA reagents, and Dr S Gillis for generous supply of TNF-R cDNA.

**References**

ADERKA D, ENGELMANN H, HORNIK V, SKORNICK Y, LEVO Y, WALLACH D AND KUSHTAL G. (1991). Increased serum levels of soluble receptors for tumor necrosis factor in cancer patients. *Cancer Res*., **51**, 5602–5607.

ALONSO S, MINTY A, BOURLET Y AND BUCKINGHAM M. (1983). Comparison of 3 actin coding sequences in the mouse: Evolutionary relationships between the actin gene of warm-blooded vertebrates. *J. Mol. Evol*., **167**, 77–101.

BISHOP JM. (1991). Molecular themes in oncogenesis. *Cell*, **64**, 235–248.

BRAUN L, DÜRST M, MIKUMO R AND GRUPPUSO P. (1990). Differential response of non-tumorigenic and tumorigenic human papillomavirus type 16-positive epithelial cells to transforming growth factor $\beta_1$. *Cancer Res*., **50**, 7324–7332.

BROCKHAUS M, SCHOENFELD H-J, SCHLAEGER E-J, HUNZIKER W, LESSLAUER W AND LOETSCHER H. (1990). Identification of two types of tumor necrosis factor receptors on human cell lines by monoclonal antibodies. *Proc. Natl Acad. Sci. USA*, **87**, 3127–3131.

CROWE PD, VANARSDALE TL, GOODWIN RG AND WARE CF. (1993). Specific induction of 80-kDa tumor necrosis factor receptor shedding in T lymphocytes involves the cytoplasmic domain and phosphorylation. *J. Immunol*., **151**, 6882–6890.

ENGELMANN H, ADERKA D, RUBINSTEIN M, ROTMAN D AND WALLACH D. (1989). A tumor necrosis factor-binding protein purified to homogeneity from human urine protects cells from tumor necrosis factor toxicity. *J. Biol. Chem*., **264**, 11974–11980.

ENGELMANN H, NOVICK D AND WALLACH DL. (1990). Two tumor necrosis factor-binding proteins purified from human urine: Evidence for immunological cross-reactivity with cell surface tumor necrosis factor receptors. *J. Biol. Chem*., **265**, 1531–1536.

GRAY PW, BARRETT K, CHANTRY D, TURNER M AND FELDMANN M. (1990). Cloning of human tumor necrosis factor (TNF) receptor cDNA and expression of recombinant soluble TNF-binding protein. *Proc. Natl Acad. Sci. USA*, **87**, 7380–7384.

HOWLEY PM. (1991). Role of the human papillomaviruses in human cancer. *Cancer Res*., **51**, 5019s–5022s.

JABLONSKA S, MAJEWSKI S AND MALEJCZYK J. (1989). HPV infection and immunological responses. In *Genitoanal Papilloma Virus Infection*, von Krogh G, Rylander E. (eds) pp. 289–331. Conpharm AG: Karlstad, Sweden.

JENSON B AND LANCASTER WD. (1990). Association of human papillomavirus with benign, premalignant and malignant anogenital lesions. In *Papillomaviruses and Human Cancer*, Pfister H. (ed.) pp. 11–43. CRC Press: Boca Raton, FL, USA.

KIVIAT NB AND KOUTSKY LA. (1993). Specific human papillomavirus types as the causal agents of most cervical intraepithelial neoplasia: implications for current views and treatment. *J. Natl Cancer Inst*., **85**, 934–935.

KOHNO T, BREWER MT, BAKER SL, SCHWARTZ PE, KING MW, HALE KK, SQUIRES CH, THOMPSON RC AND VANNICE JL. (1990). A second tumor necrosis factor receptor gene product can shed a naturally occurring tumor necrosis factor inhibitor. *Proc. Natl Acad. Sci. USA*, **87**, 8331–8335.

KRAMER SM AND CARVER ME. (1986). Serum-free *in vitro* bioassay for the detection of tumor necrosis factor. *J. Immunol. Methods*, **93**, 201–206.

LANTZ M, GULLBERG U, NILSSON E AND OLSSON I. (1990). Characterization *in vitro* of a human tumor necrosis factor-binding protein: a soluble form of a tumor necrosis factor receptor. *J. Clin. Invest*., **86**, 1396–1402.

MAJEWSKI S, HUNZELMANN N, NISCHT R, ECKES B, RUDNICKA L, ORTH G, KRIEG T AND JABLONSKA S. (1991). TGFβ-1 and TNFα expression in the epidermis of patients with epidermodysplasia verruciformis. *J. Invest. Dermatol*., **97**, 862–867.

MALEJCZYK J, MALEJCZYK M, URBANSKI A, KÖCK A, JABLONSKA S, ORTH G AND LUGER TA. (1991). Constitutive release of IL-6 by human papillomavirus type 16 (HPV16)-harboring keratinocytes: a mechanism augmenting the NK-cell-mediated lysis of HPV-bearing neoplastic cells. *Cell. Immunol*., **136**, 155–164.

MALEJCZYK J, MALEJCZYK M, KÖCK A, URBANSKI A, MAJEWSKI S, HUNZELMANN N, JABLONSKA S, ORTH G AND LUGER TA. (1992). Autocrine growth limitation of human papillomavirus type 16-harboring keratinocytes by constitutively released tumor necrosis factor-α. *J. Immunol*., **149**, 2702–2708.

MALEJCZYK J, MALEJCZYK M, MAJEWSKI S, BREITBURD F, LUGER TA, JABLONSKA S AND ORTH G. (1994). Increased tumorigenicity of human papillomavirus type 16-harboring keratinocytes is associated with resistance to endogenous tumor necrosis factor-α-mediated growth limitation. *Int. J. Cancer*., **56**, 593–598.

NOPHAR Y, KEMPER O, BRAKEBUSCH C, ENGELMANN H, ZWANG R, ADERKA D, HOLTMANN H AND WALLACH D. (1990). Soluble forms of tumor necrosis factor receptors (TNF-Rs): the cDNA for the type I TNF-R, cloned using amino acid sequence data of its soluble form, encodes both the cell surface and a soluble form of the receptor. *EMBO J*., **9**, 3269–3278.

PFISTER H. (1990). Immunobiology of papillomaviruses and prospects of vaccination. In *Papillomaviruses and Human Cancer*, Pfister H. (ed.) pp. 239–251. CRC Press: Boca Raton, FL, USA.

PORTEU F AND NATHAN C. (1990). Shedding of tumor necrosis factor receptors by activated human neutrophils. *J. Exp. Med*., **172**, 599–607.

PORTEU F, BROCKHAUS M, WALLACH D, ENGELMANN H AND NATHAN C. (1991). Human neutrophil elastase releases a ligand-binding fragment from the 75-kDa tumor necrosis factor (TNF) receptor. *J. Biol. Chem.*, **266**, 18846–18853.

SASTRE-GARAU X, SCHNEIDER-MAUNOURY S, COUTURIER J AND ORTH G. (1990). Human papillomavirus type 16 DNA is integrated into chromosome region 12q14–q15 in a cell line derived from vulvar intraepithelial neoplasia. *Cancer Genet. Cytogenet.*, **44**, 243–251.

SCHNEIDER-MAUNOURY S, CROISSANT O AND ORTH G. (1987). Integration of human papillomavirus type 16 DNA sequences: A possible early event in the progression of genital tumors. *J. Virol.*, **61**, 3295–3298.

SCHNEIDER-MAUNOURY S, PEHAU-ARNAUDET G, BREITBURD F AND ORTH G. (1990). Expression of the human papillomavirus type 16 genome in SK-v cells, a line derived from a vulvar intraepithelial neoplasia. *J. Gen. Virol.*, **71**, 809–817.

SECKINGER P, ZHANG J-H, HAUPTMANN B AND DAYER J-M. (1990) Characterization of a tumor necrosis factor α (TNF-α) inhibitor: evidence of immunological cross-reactivity with the TNF receptor. *Proc. Natl Acad. Sci. USA*, **87**, 5188–5192.

SMITH CA, DAVIS T, ANDERSON D, SOLAM L, BECKMANN MP, JERZY R, DOWER SK, COSMAN D AND GOODWIN RG. (1990). A receptor for tumor necrosis factor defines an unusual family of cellular and viral proteins. *Science*, **248**, 1019–1023.

STRAULI P, BARRETT AJ AND BAICI A. (eds) (1980). *Proteinases and Tumor Invasion*, Raven Press: New York.

WAAGE A, LIABAKK N, LIEN E, LAMVIK J AND ESPEVIK T. (1992). p55 and p75 tumor necrosis factor receptors in patients with chronic leukemia. *Blood*, **80**, 2577–2583.

WOODWORTH CD, NOTARIO V AND DIPAOLO JA. (1990). Transforming growth factors beta 1 and 2 transcriptionally regulate human papillomavirus (HPV) type 16 early gene expression in HPV-immortalized human genital epithelial cells. *J. Virol.*, **64**, 4767–4775.

ZUR HAUSEN H. (1989). Papillomaviruses in anogenital cancer as a model to understand the role of viruses in human cancers. *Cancer Res.*, **49**, 4677–4681.